# Rare case of apatinib acquired resistance induced by point mutation of WRN p.V697F through activation of the PI3K/AKT apoptosis‐inhibiting pathway

**DOI:** 10.1111/1759-7714.13726

**Published:** 2020-11-22

**Authors:** Ruofei Yu, Hua Bai, Bingyu Gao, Tangai Li, Xiran He, Pei Zhang, Jie Wang

**Affiliations:** ^1^ Department of Medical Oncology, National Cancer Center/National Clinical Research Center for Cancer/Cancer Hospital Chinese Academy of Medical Sciences and Peking Union Medical College Beijing China; ^2^ Emergency Department Peking University First Hospital Beijing China; ^3^ Dalian Medical University Dalian China

**Keywords:** Liquid biopsy, lung cancer, targeted therapy, WRN; apatinib

## Abstract

Targeted therapy has become the main treatment for non‐small cell lung cancer (NSCLC). Apatinib is a new antiangiogenic antitumor drug developed in China which targets vascular endothelial growth factor receptor‐2 (VEGFR‐2). We recently treated a 50‐year‐old female patient who underwent a bronchoscopic biopsy and was subsequently pathologically diagnosed with squamous cell carcinoma of NSCLC. EML4‐ALK and MINPP1 & PAPSS2‐PTEN fusions were found to be present in tumor tissue and blood. Sequential targeted therapy was commenced with gemcitabine + cisplatin, docetaxel, tegafur, gimeracil, oteracil potassium capsules + carboplatin, and other third‐line chemotherapy involving antineoplastic therapy, but unfortunately the patient showed primary drug resistance to this treatment regimen. Crizotinib was administered but was found to be ineffective. After two months of treatment, the disease had progressed and next generation sequencing (NGS) was subsequently performed. Apatinib was administered thereafter and the patient's symptoms improved after one week. Following administration for one month, CT scan revealed that the primary lung tumor lesions were significantly necrotic and they were narrowed. The patient's symptoms of coughing, phlegm production, and wheezing had also reduced. Her lung disease was under stable control 2.5 months later, but abdominal CT unfortunately revealed a suspected new nidus in the liver. A third gene mutation detection test showed that ALK and PTEN genetic mutations were obviously decreased; however, the patient was found to have developed WRN p.V697F (c.G2089T) point mutation, which was a new gene mutation. We suspected that the WRN gene mutation had led to apatinib resistance. We determined the absolute position of this point mutation to be chr8:30969131 with a transcript number of NM_000553.4. We retrieved information on human somatic cells from the ExAC, 1000 Genomes Browser, ESP database and PubMed databases. All the results indicated that the mutation identified in this study has not been previously reported worldwide.

## Graphical abstract

Our research group recently treated a 50‐year‐old female patient with multiple chemotherapy‐resistant, poorly differentiated lung squamous cell carcinoma. After multiple treatment attempts and regression events, the patient was treated with apatinib, which resulted in the development of a novel mutation and treatment resistance. We believe that this report will be of interest to the readership because it expands the known repertoire of mutations that can occur in lung cancer after various treatments, particularly following treatment with apatinib.

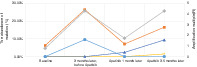

## Introduction

Lung cancer represents one of the most common malignancies worldwide, with the highest incidence and mortality, and is also considered as a lethal disease that poses a serious threat to human health.^1^ China is a country with a high incidence of lung cancer, and for nearly a decade lung cancer has accounted for the highest malignancies reported in men (first) and women (second), the rate of which increases with age.^2^, ^3^ Until now, targeted therapy has been the main treatment for non‐small cell lung cancer (NSCLC) in middle and advanced stages because it has the least side effects and the best treatment effect. The resistance to targeted therapy in lung cancer has therefore become an urgent problem in clinical research. In order to overcome the resistance to targeted therapy, third‐generation EGFR‐TKI targeted therapy has now been clinically developed,^4^ and third‐generation EML4‐ALK fusion mutation targeted therapy is now available. Apatinib is a newly developed antiangiogenic targeted antitumor drug to the market following research and development which has been undertaken in China.^5^, ^6^ It targets the vascular endothelial growth factor receptor‐2 (VEGFR‐2) of tumor cells, which can selectively compete for the ATP receptor of intracellular VEGFR‐2, blocking downstream signal transduction pathway intracellularly thereby inhibiting tumor angiogenesis. Furthermore, it has been approved for using as a second‐ or third‐line therapy in patients with non‐small cell lung cancer (NSCLC) in China, and has been found to be clincially beneficial to many patients.

**Table 1:** Liquid biopsy and next‐generation sequencing (NGS) were used to monitor the changes of tumor genome with treatment.

Tumor‐specific mutation	First treatment (baseline)	Second treatment (three months later, before apatinib)	Third treatment (apatinib one month later)	Fourth treatment (apatinib 3.5 months later)
AKT2 gene amplification	—	1.6 times		
ALK EML4‐ALK fusion	6.3%	26.5%	7.1%	16.6%
PTEN				
MINPP1 & PAPSS2‐	4.7%	25.8%	10.4%	25.8%
PTEN fusion				
ROS1 p.F339S				1.5%
WRN p.V697F			2.4%	9.4%f

**Figure 1:**
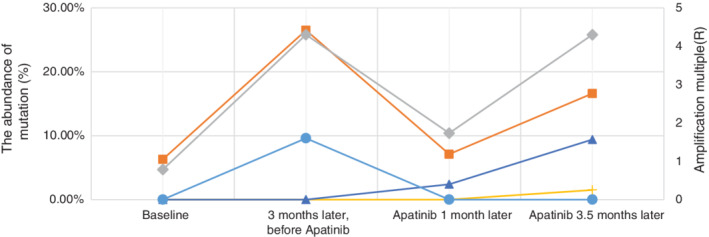
ctDNA gene expression. ALK EML4‐ALK fusion; MINPP1&PAPSS2; ROS1 p.F339S; WRN p.V697F; AKT2 gene amplification.

**Figure 2:**
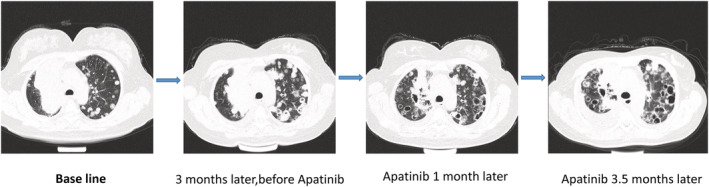
Patient tumor genome belong to a 50‐year‐old female patient with the primary multi‐resistant poorly differentiated lung squamous cell carcinoma. The first time is the situation of the tumor genome when the patient was diagnosed with lung cancer; The second time is the situation, in which the third‐line chemotheraphy and Crizontinib were in effective, but Apatinib was unused; The third time is situation that tumor genome after a 2‐month treatment of Apatinib; The forth time is the situation, in which the disease definitely progressed after a treatment with Apatinib more than 3 months. 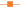, ALK EML4‐ALK fusion; 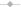, MINPP1&PAPSS2; 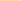, ROS1 p.F339S; 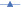, WRN p.V697F; 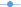, AKT2 gene amplification.

**Figure 3:**
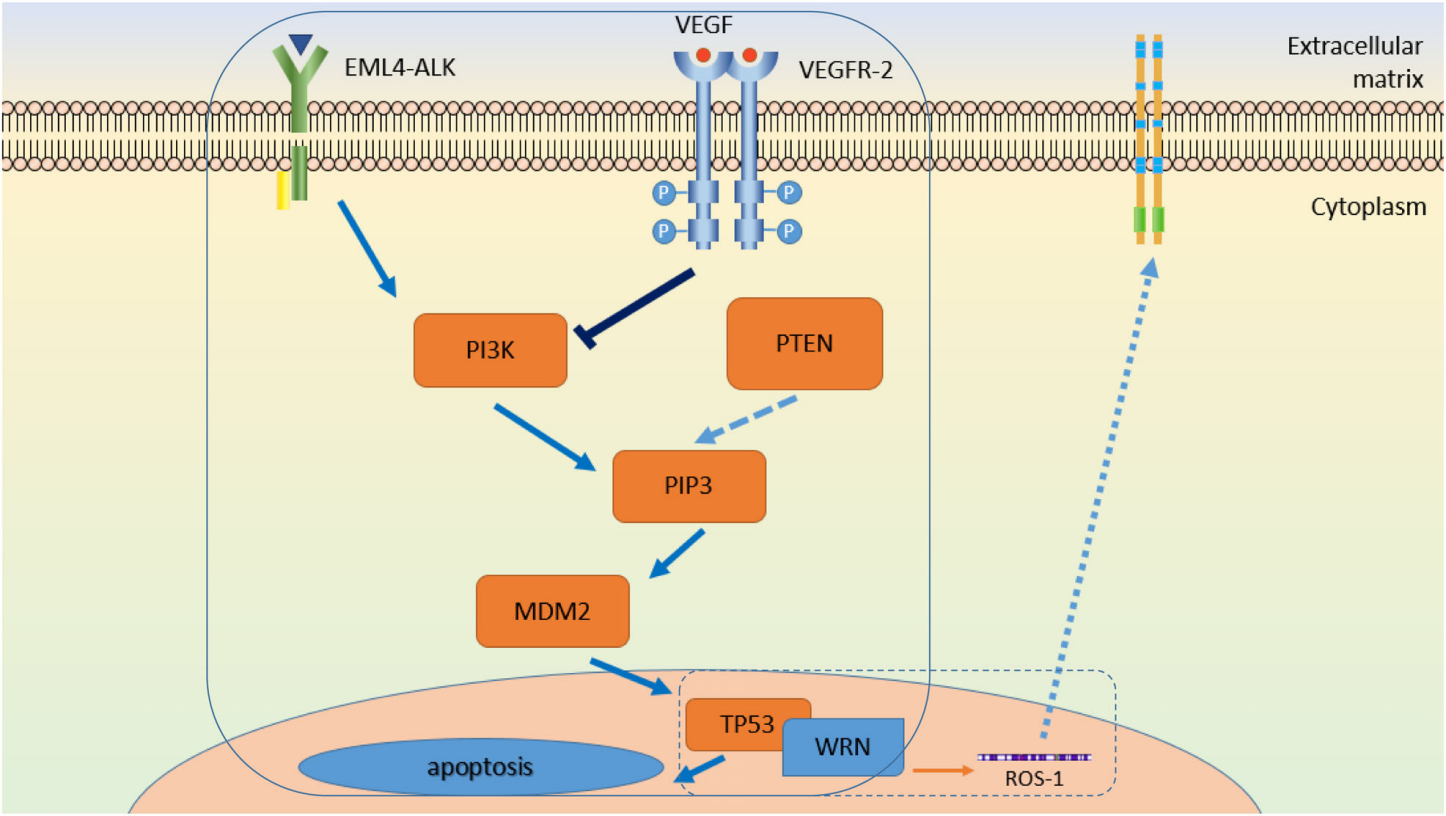
Hypothesis of Apatinib acquired drug‐ resistance mechanism caused by WRN point mutation. It is known that Apatinib inhabits the mutations of ALK and Pten, which activates the PI3K pathway, thus induced the apoptosis of tumor: we put forward a hypothesis, the binding rate of RQC structural domain in WRN gene and TP53 increased, which leads to the inactivation of TP53 again, then the apoptosis of tumor cell is controlled and the growth of tumor is promoted, thus the drug resistance appears, which is the unknown part.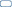, Known; 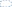, Unknown.

The research on drug‐resistant genes is restricted by technical limitations. At present, the most commonly used technology is to obtain tissue samples after a second biopsy in patients, and then first‐generation sequencing technology is applied to perform sequencing analysis site by site. However, the cost is high, the acquisition of tissue samples are difficult, detection speed is slow and efficiency is poor. However, ground‐breaking platform technology to study drug resistance is used, tumor cell lines are often included in the study, drug stimulation with median lethal concentration is given continuously until the tumor cell lines are stimulated and cultured into drug‐resistant cell lines, and NGS is then performed to study drug‐resistant mutation sites. This method has a long cycle, high reagent consumption, and additionally, is unable to reflect new tumor metastasis and other actual drug resistant processes clinically.^7^, ^8^, ^9^ Liquid biopsy is a noninvasive in vitro means of blood detection which can monitor the tumor, circulating tumor cells (CTCs) and pieces of circulating tumor DNA (ctDNA) released into the blood by metastasis. CTCs can be obtained in the blood circulation system or the tumor genomic DNA fragment ctDNA, through high‐ throughput sequencing, point mutation, fusion gene, insertion and deletion, copy number variation, chromosome rearrangement and other genetic information of the whole tumorous genome.^10^ With the development of liquid biopsy, gene sequencing can be used to study common drug resistant gene mutations, and also discover rare gene mutations which have not been previously reported, or remain unknown in academic circles. These rare gene mutations have a significant impact on the pathogenesis, resistance and progression of tumors.

## Case report

A 50‐year‐old female patient with multiple chemotherapy‐resistant, poorly differentiated lung squamous cell carcinoma presented to the clinic. This patient had previously been pathologically diagnosed with non‐small cell lung cancer by bronchoscopic biopsy with poorly differentiated squamous cell carcinoma. Next generation sequencing (NGS) was performed in lung tumor tissues, simultaneously, the second generation of venous blood was sequenced, and thus we were able to identify the original genome of the patient's tumor. NGS results revealed that both EML4‐ALK fusion and MINPP1 and PAPSS2‐PTEN fusion were present in the patient's tumor tissue and ctDNA in blood. The patient was sequentially treated with gemcitabine + cisplatin, docetaxel, S1 (tegafur, gimeracil and oteracil potassium capsules) + carboplatin and other third‐line chemotherapy of antineoplastic therapy, all with primary drug resistance. Subsequently, crizotinib, an ALK‐TKI inhibitor, was given to the patient, with poor efficacy. After two months of treatment, the disease progressed, and second generation liquid biopsy gene sequencing was performed. However, the EML4‐ALK fusion and abundance of MINPP1 and PAPSS2‐PTEN fusion in the patient continued to increase. We administered apatinib, a domestic antiangiogenesis targeted drug, and after a week of administration, the patient's symptoms had improved. A month after initial administration, CT re‐examination Figure 2 revealed that the primary lung tumor lesions of the patient were significantly necrotic and narrowed, all the intrapulmonary metastases were shown to be necrotic and narrowed, and clinical assessment confirmed partial remission (PR), with definite therapeutic effect. The patient obtained a gratifying therapeutic effect, and her cough, phlegm and wheeze were greatly reduced. The patient continued with apatinib for antitumor treatment, and thus there were few obvious side effects such as proteinuria, hypertension and bleeding. The condition of the patient was controlled and her quality of life had significantly improved. The patient continued with the treatment regimen for approximately two and a half months, and when she returned to the hospital for re‐examination, her lung disease was almost under stable control, despite the fact that abdominal CT had revealed a suspected new nidus in the liver. Considering the patient's lung disease was in a stable condition and there were few obvious symptoms, we provided apatinib for antitumor therapy. A third venous blood ctDNA gene mutation detection test was performed, and we determined that *ALK* genetic mutations and abundance of PTEN genetic mutations had obviously decreased; however, the patient was found to have developed a new genetic mutation WRN p.V697F(c.G2089T) point mutation. Therefore, it was reasonable to assume that the occurrence of the mutation in the WRN gene had led to apatinib resistance in this patient. The patient continued to require oral path for targeted antitumor treatment, and a month later, the patient's condition had obviously progressed. A CT scan revealed that there was brain and liver metastasis, and this was the fourth time of detection. The types of WRN p.V697F(c.G2089T) point mutation had obviously increased. At the same time, a new ROS1 p.f339s point mutation had appeared, so it was reasonable to infer that under the pressure of apatinib, the mutation had produced a proliferation of new gene mutation subclones. Hence there are many possibilities as to the appearance of the ROS1 mutation Table 1.

## Discussion

Following statistical analysis undertaken in our hospital and cooperation with northeast China and other regions with regard to tumor genome sequencing data and preliminary studies, we determined that the mutation probability of the WRN gene is low, and to date that its function had not been clearly studied and elucidated.

The WRN gene is located at chromosome 8 in human cells (8p12), and was first located by the linkage analysis technique in 1992.^11^ In 1996, the WRN gene was identified by a traditional localization cloning method and sequenced. The decreased activity of WRN gene promoter is associated with autosomal recessive genetic disease in adults with premature ageing, known as Werner syndrome (WS). The WRN gene spans more then 250 kb and consists of 35 exons, of which there are 34 coding exons. The code is composed of 1432 amino acid residues, and the WRN protein with a molecular mass of 160 kD_RecQ3, is considered as the most important of the five RecQ helicases in the human body. It can maintain genome stability, play a significant role in the multiple DNA damage repair pathway and inhibit the occurrence of premature aging and tumors.^12^ It contains a highly conserved RecQ C‐terminal region (RQC), which can directly bind to a large number of tumor‐related proteins such as BRCA1, TP53, etc. WRN have also been reported to bind to TP53 through the RQC structural domain.^13^, ^14^ TP53 inhibits the activity of the WRN helicase,^14^, ^15^ TP53‐dependent apoptosis in cells is also impaired,^16^, ^17^ and thus plays an important role in tumorigenesis and tumor development. At present, the polynucleotide polymorphism of the WRN gene has been reported to be closely related to breast cancer in Chinese females, prostate cancer, esophageal cancer and non‐Hodgkin's lymphoma.^18^, ^19^, ^20^, ^21^, ^22^ It has also been reported that individual point mutations in WRN have been found to be associated with the respond intensity to chemosensitivity.^19^, ^20^ In this study, we found that the apatinib resistant gene in a patient with multidrug‐resistant, poorly differentiated squamous cell carcinoma is WRN p.V697F(c.G2089T) point mutation. This point mutation uses ENSEMBL database and GRch37/HG19 is considered as a reference genome. The absolute position of this point mutation is chromosome chr8:30969131, the transcript number is NM_000553.4. The human somatic cell was retrieved from ExAC, 1000 Genomes Browser, ESP and Pubmed databases. All these results indicate that this genetic mutation is a new mutation, which has not as yet been reported worldwide. Therefore, WRN mutation may be a possible drug resistance mutation to apatinib.

In the pathophysiology of human NSCLC, the expression of EML‐ALK fusion‐type oncogenes have been reported to increase, activating intracellular tyrosine kinases, phosphorylating their downstream substrates and promoting the signaling pathway of PI3K/AKT in tumors.^23^ In other words, EML‐ALK fusion‐type oncogenes activate PI3K and upregulate downstream PIP3, MDM2, which causes the negative regulation of TP53 decrease and thus inhabits and induces apoptosis in tumor cells. Meanwhile, the fusion mutation of MINPP1 & PAPSS2‐PTEN causes the decrease in the protein phosphorylase and esterase in the PTEN gene,^24^ thus inducing the antagonistic inhibition of the PI3K/AKT signaling pathway and promoting the upregulation of PIP3, while the receptor of VEGFR2 blockades the downstream signaling pathway of PI3K, promotes the original activation pathway, thus upgrading TP53 and inducing tumor apoptosis. Previous studies on the WRN gene are based on the mechanism of physiological or tumor pathophysiological progression, whereas there are no studies or reports on the mutation of drug resistance.

Therefore we hypothesized that due to the suppressive effect of apatinib, the point mutation of WRN p.V697F(c.G2089T) might be responsible for inducing gene activation, thus the binding rate of RQC structural domain in WRN gene and TP53 increased, which again led to inactivation of TP53. Tumor cell apoptosis is thereby controlled and tumor growth is promoted, thereby leading to drug resistance. Whether the WRN gene induces acquired resistance to the targeted drug apatinib due to this mechanism should be elucidated in future studies Figure 3.

## Disclosure

The authors declare that there are no conflicts of interest.
